# How, When and Where to Discuss Do Not Resuscitate: A Prospective Study to Compare the Perceptions and Preferences of Patients, Caregivers, and Health Care Providers in a Multidisciplinary Lung Cancer Clinic

**DOI:** 10.7759/cureus.257

**Published:** 2015-03-12

**Authors:** Naseer Ahmed, Michelle Lobchuk, William M Hunter, Pam Johnston, Zoann Nugent, Ankur Sharma, Shahida Ahmed, Jeff Sisler

**Affiliations:** 1 Department of Radiation Oncology, CancerCare Manitoba, University of Manitoba, Canada; 2 Faculty of Nursing, University of Manitoba, Canada; 3 Radiation Oncology, Tom Baker Cancer Centre; 4 Department of Nursing, Cancer Care Manitoba, Canada; 5 Department of Epidemiology and Cancer Registry, Cancer Care Manitoba, Canada; 6 Cancer Care Manitoba, University of Manitoba, Canada; 7 Radiation Oncology, Cancer Care Manitoba, University of Manitoba, Canada; 8 Family Medicine, University of Manitoba, Canada

**Keywords:** dnr, lung cancer

## Abstract

*Background:* Do Not Resuscitate (DNR) is a significant but challenging part of end-of-life discussions when dealing with incurable lung cancer patients. We have explored the perceptions and preferences of patients, their caregivers (CGs), and health care providers (HCPs) and the current practice and opinions on DNR discussions in a multidisciplinary lung cancer clinic.

*Materials and Methods:* This is a prospective descriptive study with a mixed quantitative and qualitative methodology to capture perceptions of the participants. To obtain a rich description of participant responses to questionnaire items, we employed a ‘think aloud’ process that prompted participants to immediately verbalize their thoughts when responding to questionnaire items. We used content analysis and constant comparison techniques to identify, code and categorize primary themes in the captured data.

*Results*: Ten patients with advanced-stage lung cancer; nine CGs from the lung clinic and ten HCPs from the Thoracic Disease Site Group (DSG) were enrolled in the study. Most patients had only a limited understanding of DNR. Most CGs had a fair to good understanding of DNR. Most HCPs perceived their patients to have understood DNR most of the time. When patients were interviewed, a theme of “anticipated discussion” about DNR was identified. Patients and CGs expressed having faith in the system and responsible physicians as to when to discuss DNR. HCPs embraced a clinician preference-based decision-making approach to engaging in DNR discussions. They desired *more resources*, *more knowledge*, *more structure* and *more time* to discuss DNR. Most HCPs felt that it would be worth conducting a prospective clinical trial to determine the best time to discuss DNR.

*Conclusions*: This pilot study provides a unique mixed quantitative and qualitative understanding of the perceptions of patients with lung cancer and their CGs and HCPs regarding DNR discussion. Our findings will help further the development of evidence-based guidelines and a broad prospective study that would have important implications for policies and practices around DNR discussions in order to reduce the emotional pain of dying patients, their CGs and HCPs.

## Introduction

Most patients with lung cancer are found to have incurable diseases shortly after their diagnosis. More than one million people in the world die of this disease every year [1]. While patients struggle to reconcile themselves with the reality of impending death, the families, nurses and oncologists involved in their care are faced with the challenge of finding the appropriate timing and environment to initiate End of Life (EOL) care discussions. Do Not Resuscitate (DNR) is the most significant and critical part of such discussions. One of the most commonly cited reports involving more than 9,000 patients with life-threatening diseases revealed that only 47% of physicians knew the DNR status of their patients and 46% of DNR orders were written within two days of death [2]. Given the current global awareness and cultural shift towards patient autonomy, patients desire to know their prognosis and be a part of the decision-making process: a caring and sensitive physician is an integral part of such communication [3-5]. When DNR is not addressed in advance, terminally ill hospitalized patients may undergo unwanted interventions [6].

Cancer Care Manitoba (CCMB), Canada is a tertiary care center with a well-established multidisciplinary Thoracic Disease Site Group (DSG) that sees approximately 800 newly-diagnosed lung cancer patients per year. Upon being assessed as incurable, patients are often enrolled in the palliative care program at CCMB. This is a comprehensive program intended to address the multidimensional needs of patients and their families. One of the most important pre-requisites for patients to enroll in this program is to have their DNR status determined and signed by an authorized cancer care provider which, in most cases, is either an oncologist, a physician designated by the oncologist, or a nurse practitioner with special training and experience in the field of oncology. Based on the evidence from an extensive literature review and our clinical experiences, this discussion can at times cause significant distress to the patient, their family and cancer care providers involved in this discussion [7]. Members of CCMB Thoracic DSG ,including oncologists, nurses, palliative care physicians and psychosocial counsellors, identified a need to explore the current practice and opinions around DNR discussion and future development of advance care planning guidelines, which forms the basis of this study.

We aim to present perceptions and preferences of patients with incurable lung cancer, their family caregivers (CGs), and their health care providers (HCP) involved in the care of lung cancer patients, followed by another paper where we present more in-depth analysis of perceptions and preferences of patients and their CGs in response to DNR discussions. 

## Materials and methods

The study was approved by the Research Ethics Board University of Manitoba (approval #H2011:227). Informed consent was obtained for this study.

### Sample size and recruitment of participants

Patients and CGs

We aimed to recruit from the lung cancer clinic at CCMB, a purposive sample of ten patients and ten CGs. The eligibility criteria for patients included individuals who: received a pathological diagnosis of non-small cell and small cell carcinoma, had incurable disease, and were not to receive further curative therapy. In addition, patients may have either accepted or declined a DNR order following a discussion of their DNR status with their HCP. Patients were able to attend a CCMB clinic or be seen on a hospital ward to complete the interview and have a CG who was a willing study participant. The inclusion criteria for CGs included individuals who were: nominated by the patient as the primary adult individual who assisted them with his or her care, involved in the DNR status decision-making with the patient, and able to attend a CCMB clinic or be seen on a hospital ward to complete the interview.

Members of the Thoracic DSG identified potential patient participants who met the inclusion criteria. A designated research nurse then invited eligible patients and CGs to participate in the study. If the patient and the CG agreed to participate, then the research nurse scheduled separate face-to-face interviews with them at CCMB or on a hospital ward. Informed consent was obtained by the research nurse from all the participants prior to conducting the interviews.

HCPs

We recruited ten HCPs who met the eligibility criteria described as any physician, a nurse, or a psychosocial counselor who was a member of the CCMB Thoracic DSG. The designated research nurse invited eligible HCPs to participate in the study. Informed consent was obtained by the research nurse from all participants prior to conducting the interviews.

### Design and administration of the study

We employed a mixed quantitative and qualitative methodology to capture perceptions of the participants. In order to adhere to a consistent interviewing technique and maintain the quality of audio-recorded interviews with study participants, we provided training on the interview technique to the research nurse who was designated to conduct all participant interviews.

Quantitative Component

Patients, CGs, and HCPs were asked to complete respective pen-and- paper versions of an investigator-developed questionnaire. Socio-demographic and medical information of patient participants were collected from patients and verified with CCMB medical records (see Appendices 1-3).

Qualitative Component 

To obtain a rich description of participant responses to questionnaire items, we employed a ‘think aloud’ process where the research nurse prompted participants to immediately verbalize their thoughts when responding to questionnaire items on DNR status and DNR discussions. These prompts explored their level of understanding, personal wishes, triggers, timing, appropriate setting, emotional experiences, appropriate health care provider, and the extent of family dialogue on DNR decisions (see Appendices 1-3). The research nurse was guided by an open interview script with prompts. Interviews with participants were audio-recorded and transcribed for interpretation and analysis of the key themes. The in-depth responses provided by respondents precluded reporting all data in one manuscript. Hence, in this article, only portions of qualitative responses to interview questions are presented. The second manuscript will provide greater detailed information from the patient and family perspective based on their experiences, beliefs, and preferences.

### Data analysis

Descriptive statistics were used to describe the demographic and medical characteristics and subgroup responses to the questionnaire items.

Qualitative Component

The audiotapes of participant ‘think aloud’ responses to questionnaire items and semi-structured interview questions were transcribed by a trained medical transcriptionist. The investigators then examined the transcripts and used content analysis and constant comparison techniques to identify, code and categorize primary themes in the data [8].

Themes and Coding

Three members of the investigative team (NA, WH and ML) extracted possible themes from transcribed data from three groups of participants. All themes were subsequently discussed and consensus was obtained for the extracted themes. A coding template was developed for each of the themes and served the basis of ongoing analyses and interpretation of the remaining transcripts by the third author. Regular meetings occurred between the first and third authors to discuss the addition or revision of themes as data analyses and interpretation ensued. 

## Results

### Participant characteristics

Ten patients with advanced stage lung cancer, nine CGs and ten HCPs from the Thoracic DSG were enrolled in the study. Due to logistical reasons, one CG could not be interviewed and was excluded from the analysis. Patients, CGs, and HCPs demographics are outlined in Tables 1-3. The majority of patients were male, 67.5 years of age (median), affiliated to some religion and had pathologically confirmed non-small cell lung cancer, an identified DNR status, no brain metastases, and had received no whole brain radiation prior to being interviewed. Most patient participants were interviewed in the outpatient clinic at CCMB (Table 1). The majority of caregivers were female, retired, 67.5 years of age (median), the spouse who lived with the patient, varied in religious and ethnic backgrounds, reported having less than or equal to a high school education, and earned either $40,000 to 80,000 or less than $20,000 in annual income (Table 2). The majority of HCPs were female and were either a nurse or a medical or radiation oncologist (Table 3). 

**Table 1:** Patients (n = 10) characteristics

Characteristics	Patients (n)
Median Age	67.5 years
Gender	
M	6
F	4
Religious Affiliation	
Affiliated to Some Religion	9
Not Affiliated to Any Religion	1
Histological Diagnosis of Lung Cancer	
Non-Small Cell	9
Small Cell	1
DNR Status	
DNR	9
No DNR	1
Brain Metastasis	
Present	3
Absent	7
Whole Brain Radiation Prior to Interview	
Received	2
Not Received	8
Location of the Interview	
Outpatient Clinic	8
In Hospital	2

**Table 2:** Caregivers (CGs) (n = 9) Characteristics

Characteristics	CGs (n)
Median Age	67.5 years
Gender	
M	3
F	6
Relationship	
Spouse	5
Parents	1
Sibling	2
Friend	1
Religion	
Anglican	2
Lutheran	1
Greek Catholic	1
United Church	4
None	1
Culture/Ethnicity	
Scottish, Italian	1
French, Quebec	1
Icelandic Irish	1
Irish English	1
Scottish, German	2
Ukrainian	2
Ukrainian Polish Dutch	1
Education	
High School or Less	6
University	3
Income	
40-80,000K	3
<20K	2
No Answer	2
Occupational Status	
Working Part-Time or Full- Time	3
Retired	6
Frequency of Contact with Patient	
Living with Patient	7
Visiting Weekly	2

**Table 3:** Health Care Providers (HCPs) (n = 10) Characteristics

Characteristics	HCP (n)
Gender	
M	4
F	6
Profession	
Medical Oncologist	2
Nurse	4
Psychosocial Counselor	1
Radiation Oncologist	2
Surgeon	1

### Understanding of DNR

The following describes quantitative and qualitative responses by patients, CGs, and HCPs on how patients and CGs understand DNR status (Table 4). Exemplars of study participants’ qualitative responses provide some insight as to the rationale for responding as they did. Some responses were common to all three groups and some are exclusively from HCPs.

**Table 4:** Understanding of DNR status Most patients reported they had fair to extremely limited understanding of DNR. Most CGs had a fair to good understanding of DNR. Most HCPs perceived their patients to frequently understand the meaning of DNR.

How would you rate your understanding of the concept of resuscitation status or DNR?	Patients (n = 10)	CG (n = 9)
Good	3	6
Fair	1	1
Extremely limited	1	0
Lacking in some areas	5	2
Do your patients understand DNR?	HCPs (n = 10)	
Always	1	
Frequently	7	
Sometimes	2	

When patients, CGs, and HCPs were prompted to explain their responses to their respective questions noted in Table 4, the theme of *an anticipated discussion* was found.

*An anticipated discussion* describes expressions by patients and their CGs on how they had previously thought about the meaning of DNR within the context of their preferences and goals for ongoing care that was evident to their HCPs. Most patients demonstrated their awareness of the meaning of DNR and desired medical attention for reversible and irreversible conditions. For instance, one patient stated,

“I want them to keep me comfy and beyond that I don’t want them to put me on machines and what not,” and another patient described, “So if it was something minor … but if it was bronchitis or something and they could give you an antibiotic yes, I mean I would want that.” 

Often patients’ responses were in relation to describing their specific desires while considering the degree of seriousness of an imagined or future medical state. On the other hand, CGs expressed their awareness of the extent and content of information shared by health care providers about what DNR means for the patient. CGs appeared to be more inclined to provide a simple definition of DNR status. They did elaborate on different patient medical states that may require varied degrees of medical intervention or described preferences by patients for medical attention. One caregiver stated,

“It means to me, in serious complications with the prognosis of her health that she would not be artificially kept alive to sustain her status if the conclusion of the medical people is such as the case.”

Many HCPs described that most patients and CGs had already thought about the meaning of DNR in anticipation or preparation of having this discussion with their HCP. In other words, most HCPs shared their perceptions that patients had already entertained thoughts about having a poor prognosis prior to a discussion with their HCP about DNR and preferences for medical attention. One HCP (oncologist) stated:

“But I think the nature of the disease that I treat is lung cancer mainly, and you know most of the people are diagnosed at an advanced stage, they know death is the inevitable event out of this cancer, so usually they anticipate that when we talk about DNR. I very rarely have people that didn’t understand what it does so I don’t think it was a major issue in my practice.”

Another HCP (nurse) described,

“I think they often do. The idea that if you have end-stage disease and you’re on the verge of multi-system failure that, you know.”

HCPs were also prompted to explain how they knew that their patients understood or did not understand DNR when discussed. Some HCPs explained that, due to their direct explanation and clarification of what to expect, they felt satisfied that their patients and CGs understood DNR. The following statement provided by one HCP demonstrated his or her belief in the key role that the HCP has to play in taking a direct approach by providing a clear explanation of outcomes of treatment and next logical plan of care:

“Oh I directly ask them, I don’t go around it… Sometimes I have spoken about it even before that.” Another HCP similarity stated: “And I feel like I would assume that by the end of our conversation they would understand it because I would normally check out what they understand, right”.

The above statement indicates how he or she valued clear, direct communication and validation of whether the patient and CG understood the implications of DNR. Still another HCP described an appreciation for gauging patients’ understanding of DNR based on the questions they ask and their prompts for more information about DNR: *“Because of the questions they ask for clarification.”*

### Appropriate time to discuss DNR

Patients, CGs, and HCPs were asked ‘when’ was the most appropriate time to discuss DNR. Their quantitative responses are outlined in Table 5.

**Table 5:** Appropriate time to discuss DNR Patients and CGs described that the most appropriate time to discuss DNR was either at the time of initial diagnosis, when the disease is considered incurable, or when the patient is being transferred to palliative care. Most HCPs considered the appropriate time to discuss DNR was when patients were informed of poor prognosis and/or referred for palliative care.

Appropriate time to discuss DNR	Patients (n = 10)	CG (n = 9)
At time of initial diagnosis	3	2
On follow up visit after previously being told of poor prognosis	1	1
On hospital/palliative care admission	1	1
Only when death is imminent	1	
Other	1	1
When discussing referral to palliative care program	1	1
When poor prognosis is first heard	2	3
Appropriate time to discuss DNR	HCP (n = 10)	
Discussing referral to palliative care	2	
On follow up visit after previously being told of poor prognosis	1	
Other (at any time, patients may have opinions even when well)	1	
Other (depends)	1	
When first told of poor prognosis	5	

To further explain their responses to the timing of having a DNR discussion when prompted during interview, the following theme of *“Having faith in the doctor as to when to discuss DNR” *was captured. Most patients expressed ‘faith’ in their doctor’s discretion. Essentially, patients described that the physician was responsible to identify the appropriate time and opportunity to initiate the DNR discussion with the patient. One patient stated:

“I think once they say or Dr. X [oncologist] says “I can’t do anymore, we’re going to refer you on to a Palliative Care doctor and nurse and so on,” that sort of tells me that you know the DNR should be discussed now.”

Similarly, most CGs described having a faith in the health care system and the physician’s discretion to engage in a timely discussion about DNR. For example, one CG stated,* “Well because again, we put our faith in the health system*.” Having this expressed faith in the health care system appeared to be based on the skilled knowledge of HCPs that, however, was not directly elucidated by patients and CG participants.

The requisite skills and knowledge were described by one HCP who shared his or her experience with challenges and sensitivities related to the need to respect patients’ hope and psychological status, which guided the timing of a DNR discussion:

“It’s always a balance between keeping the hope going for those people versus getting things ready for them…But there is a huge factor in psychological impact and how people cope and quality of life and all that so I cannot go by this technicality and say you have an incurable disease and we need to talk about DNR today. So it’s always a difficult balance.”

As well, some HCPs felt that knowing when to engage in the DNR discussion involved a delicate balancing act, which is impacted by the unique situation or condition of the patient. For instance, one HCP shared:

“It depends on a lot of things… [provided scenarios] … so I can’t say that any specific circumstances would be the best time,” and “It’s based on what’s their need for information and when does that come up? It’s very situational. So it could be appropriate any of those times."

On the other hand, other HCPs carefully watched for signposts as to when to discuss DNR with patients that included before receiving palliative care or when patients explicitly expressed no desired ‘heroics’ in their care. For instance, several HCPs stated:

“… it definitely has to happen before the Palliative Care referral … if you discuss the DNR at that time then you are reasonably safe,” and “There’s one where I think it says 'where death is imminent' … You may have somebody who comes in and from the get go is saying, 'I want no heroics' … So that could be another point?"

When HCPs were asked if they felt comfortable discussing DNR, all ten participants were affirmative in feeling at ease in leading a discussion with patients about DNR. Based on HCPs’ responses on ‘timing,’ it appeared that individual HCPs embraced a clinician preference-based decision-making approach, thus making it more comfortable for them to engage in DNR discussions with their patients. (I.e., either basing the DNR discussion on signals they identified from the patient’s unique situation or preferences, unfolding treatment phases that moved from curative to palliative treatment or the patient’s explicit wishes.)

In the following, we exclusively present responses by HCPs when asked about aspects of DNR practices, policy, and the workplace or environment that required improvement and further study under the major theme of *“Revisiting current practice.*” Seven out of 10 HCPs stated that they thought that current practice and the policy of DNR in their workplace required revisiting. To further explain their responses under "*Revisiting current practice*," several sub-themes identified specific elements that require further attention, including the need for *“more resources,*” *“more knowledge*” and *“more structure”* in the clinic setting. 

### “More resources” 

The sub-theme of *more resources* captures recommendations shared by some HCPs about workplace elements that are needed to bolster their ability and/or confidence to engage in DNR discussions. For instance, the need for more or protected time to engage in difficult DNR discussions was a key resource expressed by six out of 10 HCPS. As one HCP (oncologist) stated:

“Because we have 15 minutes for the appointment; and, that’s the appointment we might encounter that they are not doing well and in that 15 minutes we have to talk about that then we have to talk about DNR, what does that mean, and what is it subsequent to that, and then go and dictate that and send the letters and all that. It is impossible.”

Besides the need for more time, several HCPs also felt that a dedicated, private room was needed for confidential and safe expression of feelings by patients. Of note, all ten HCPs stated that the clinic was the best setting to discuss DNR. The hospital setting was perceived as the worst place due to the lack of privacy that does not foster sensitive interaction about DNR. Other HCPs explained that the patient’s situation and other factors such as psychological status of the patient, also dictate the setting for optimal, timely dialogue and decision-making about DNR. Furthermore, all ten HCPs described that the physician, the patient, and single or multiple family members should be present during the DNR discussion to attain a shared understanding of decisions about DNR. Again, this provides additional rationale for HCPs’ expressed need for more private, comfortable and dedicated spaces for uninterrupted DNR discussions in busy clinic settings.

### “More structure”

The sub-theme of *more structure* addresses HCPs’ expressions of an organized, evidence-based approach to guide difficult DNR discussions. For instance, six out of 10 HCPs felt that a script would be useful as a template or a guide to engage in a DNR discussion with patients. One HCP stated,

“But very often when you have a script and you see how it’s put in there, then you adapt that and you can use your own words.  So having a script might be helpful in the same way. Not that the practitioners are going to use those words necessarily but it gives them a bit of a template and they can substitute their own.”

Other HCPs described the need for more formalized involvement of other health care disciplines to meet the patients’ multi-faceted needs (e.g., physical, emotional, spiritual, financial, etc.) when discussing DNR or in follow-up to address concerns with a social worker, palliative care physician or nurse practitioner:

“*More time and more personnel and nurse practitioners … it’s not that we are trying to hide away from it, but I could initiate it, but sometimes I feel that we leave for the next patient; you don’t know how they are doing … So if somebody could take that on and explain everything how that would be when they go home, they’ll feel much better.”*

### “More knowledge”

The sub-theme of more knowledge addresses HCPs’ expressions of their lack of confidence in taking an appropriate, personalized approach to engage in DNR discussions. There was one HCP, however, who indicated that oncologists are sufficiently trained to engaging in difficult DNR discussions:

“I mean, we all have trained as oncologists. If you need to teach us something, I’m talking on a physician’s point of view, I shouldn’t be here”.

Still others indicated the need for more training and evidence-based information packages to engage in difficult discussions about breaking bad news. Being skilled and confident to ‘personalize’ the DNR discussion was important to this HCP:

“There are actually guidelines … well, breaking bad news is obviously what we are talking about … there’s actually compulsory training in some institutions in Europe. All staff have to have compulsory training on breaking bad news. Even if you have been doing it for a long time you still have to go on the training course and there are guidelines for how it should be done, but also where it should be done.”

Some HCPs recognized the need for more training or awareness of how to approach the topic of DNR while considering the cultural background, beliefs, values and preferences of patients and families seen in the cancer clinic:

“There are certain races that do not really like to talk about prognosis so that makes it extremely difficult to talk about end of life issues, DNR, that sort of thing. Sometimes they don’t want whoever’s sick to hear so they really try to control what the patient gets told which makes it extremely difficult because you’re kind of being run by everybody else.”

In addition, the patient’s age, psychological impact of the prognosis and implication of DNR, rapid deterioration in the patient’s condition, religion, and language barriers are factors described by HCPs as adding complexity to their DNR discussions with patients and families.

Six out of 10 HCPs felt that it would be worth conducting a prospective clinical trial to determine the best time to discuss DNR. One HCP explained:

“I think one of the things that happens when people are doing research is regardless of the outcome of the research, in the process of the research being done, people are thinking about it.”

The above statement indicates that a trial may initiate reflection and dialogue about the issue or an appropriate time. On the other hand, four out of 10 HCPs did not feel a clinical trial was a worthwhile endeavor to answer questions about timing of DNR discussions. For instance, one HCP offered the following cautionary statement: *“Medicine is an art and a science,” *suggesting that clinical trials are not a panacea to determining the right time to hold a DNR discussion.

## Discussion

Deciding on the patient’s DNR status is vital to the care of advanced stage lung cancer patients. The patient’s DNR status influences how oncologists, family physicians, oncology nurses and social workers manage clinical and psychosocial aspects of a patient with an incurable cancer. Evidence-based recommendations concerning how to discuss DNR with patients and their families are lacking. The need to conduct this pilot study arose due to the lack of a well-defined policy to discuss DNR in an academic center where specialized HCPs provide comprehensive cancer care in an established Thoracic Disease Group. In this paper, the authors focused on describing preferences and perceptions of HCPs to discuss DNR.

A shared understanding of the concept of DNR by the patient, the CG, and their HCPs is crucial in the decision-making process. In our study, when patients were interviewed, they all expressed a limited degree of understanding of DNR. Most of the CGs had reported an adequate understanding of DNR. Almost all participating HCPs perceived their patients to understand DNR “most of the time.” A cross-sectional survey involving five Canadian teaching hospitals with 477 patients and 160 CGs revealed that most patients were not aware of the nature of the process of the Cardio Pulmonary resuscitation (CPR). Subset analysis indicated that cancer patients were more likely “unaware” (53.6%) of the key components of CPR as compared to patients with other medical conditions (38.7%). Only a very small number of patients (< 3%) had an adequate knowledge of CPR outcomes [9]. On the contrary, in another Canadian study published in 2012 that involved 429 patients (none were diagnosed with cancer) who presented to their primary care physicians, 84% of 386 respondents were aware of the term DNR. When these respondents were evaluated further, 83% of the ‘DNR aware’ patients correctly identified the definition of DNR [10]. On the other hand, this study’s quantitative and qualitative responses indicated that most of the patients with lung cancer lacked a full understanding of the DNR concept. It is difficult to understand why patients lacked a greater understanding of DNR without further prompting which is a limitation of our study.

Interestingly, HCPs perceived their patients to have understood DNR most of the time.

Studies indicate oncologists have communication difficulties and hold misperceptions and misunderstandings when discussing EOL issues with their patients [7, 11-14]. Baile stated that, *“This is a mistaken belief that patient communication is an innate skill of minor importance when compared with the technical aspects of care *[11]*.*" Further research is needed to more fully examine the rationale for discrepancies between the physician and patient/CG level of understanding of DNR.

Choosing the appropriate time and environment to discuss EOL issues, specifically about DNR, is a challenging and complex task. In this study, the quantitative responses of patients and CGs were diverse (Table 5). However, both patients and CG expressed their faith in the health care system in making this determination. Both groups felt that the physician is responsible for choosing the appropriate time or opportunity to initiate the DNR discussion. This is in concordance with published literature, as most patients believe physicians should be the one to initiate such discussions [15-16]. When HCPs were asked the same question, they offered varied responses: “when first told of poor prognosis,” and “at the time of referral to palliative care,” as the time points for DNR discussion. Further examination of qualitative responses, however, indicated that most of the HCPs described “before palliative care” as the best time to discuss DNR. A study from Australia with a similar group of participants (19 palliative care patients, 24 CGs, and 22 HPs) found the following themes when asked about who and when to initiate discussions about prognosis and end of life issues:* “wait for the patient or caregiver to raise the topic,” “health professionals (HPs) to offer all palliative care patients and their caregivers the opportunity to discuss the future,” “HPs to initiate the discussion when the patient/family needs to know,” and “HPs to initiate the discussion when the patient/family seem ready *[17]." These themes are similar to the themes expressed by the patients and CGs in our study.

Regarding the need for a script or a structured information sheet to discuss DNR, HCPs provided conflicting responses. Some HCPs, specifically oncologists, felt that oncologists received sufficient training to engage in DNR discussions without a structured script. Interestingly, other studies have shown that although physicians may be sufficiently trained to manage medical issues, they are least trained to communicate with dying patients and their families [18-19]. In his paper, “A Physician’s Guide to Talking About End-of-Life Care,” Balaban narrated a typical scenario that involves a DNR discussion led by physicians and other authorized HCPs [20]. In this scenario, very sick patients are asked questions about Cardio Pulmonary Resuscitation (CPR) in the most mechanical fashion. Patients are asked if their heart stops, do they wish to be revived with electrical shocks, chest compression, intubation, and connection to a ventilator? Such a script appears to be inadequate in many respects. EOL discussions should not be limited to DNR. HCPs need to address the psychosocial aspects of the dying patient and his family. Balaban provided a comprehensive four-step guide for physicians to discuss EOL care [20]. A review of the literature indicated that, in most situations, physicians describe the mechanics of CPR to patients with terminal cancer without discussing the overall goals of care. Physicians act on an urgent need to discuss and document the DNR status without discussing issues that are significant to the dying patient [21-22]. These issues include physical and psychosocial concerns, including the patient’s fears of pain, indignity, uncertainty, and abandonment, which are a vital part of any EOL discussion [23-25]. von Gunten, through real-time clinical experiences, has pointed out serious gaps in the process of DNR discussions with cancer patients and provided a comprehensive multi-step approach to discussing DNR [26].

Finally, all HCP participants in our study indicated that the outpatient clinic is the most appropriate place to discuss DNR. Some HCPs considered the hospital as the worst place for such discussions. As evidenced by HCP responses in this study, there is also a desire to revisit the current institutional policy and practice to discuss DNR and possibly conduct a prospective trial to build a knowledge base that describes the most appropriate time, environment, or resources, and structure of DNR discussions.

## Conclusions

Our pilot study provides a unique mixed quantitative and qualitative understanding of the perceptions of patients with lung cancer alongside their CGs and HCPs regarding DNR discussions. Our study findings lend evidence to the need for further development of evidence-based guidelines that address issues of concern raised by patients, their families and HCPs about current practices and policies regarding DNR.

The next steps in our research include a broad prospective study including patients with lung and other terminal cancers where EOL issues are frequently discussed and where the timing of DNR discussions could be evaluated empirically. Findings from such a large study would have important implications for policies and practices around DNR discussions that will benefit not only those who suffer emotional pain during the last days of their life, but also the team of dedicated oncologists, family physicians, palliative care physicians, nurses and psychosocial counselors who genuinely desire to facilitate comfort and a pain free death to the terminal cancer patients.

## Appendices

Appendix 1: Patient Survey

Part B (questionnaire and interview)

Prompt*: Today we’re going to be talking about medical issues such as advance care planning and resuscitation status, which can be a tiring and emotional experience for some. May I remind you that you are free to decline to answer any or all questions if you wish? Over the course of the interview, you and/or your caregiver may become fatigued or feel too unwell to continue the interview. If this happens, you and/or your caregiver can request the interviewer to stop the interview at any time. The interviewer will then invite you and/or your caregiver to continue the interview at another time or date that is convenient for you and/or your caregiver. Please ask for clarification if any questions are difficult to understand. For the purpose of this survey such terms as “resuscitation status,” “Do Not Resuscitate or DNR order,” and “Advance Care Plan or ACP level” may be used frequently and interchangeably.*

What is your understanding of your cancer currently?

Prompt: *a. Have your health care providers discussed the prognosis with you?*

              b. How do you feel about discussing prognosis with your health care providers?

How would you rate your understanding of the concept of “resuscitation status” or “DNR”?

1. Extremely limited
2. Lacking in some areas
3. Fair
4. Good
5. Excellent

Prompt: *Please describe your understanding of resuscitation status or DNR in your own words. *

Prompt*: I wish to understand your own personal wishes and desires about DNR.*

What are your own wishes in the event that your heart and lungs stop?

1. Can you further share your wishes about attempts being made to keep you alive if your heart or breathing stops with things like breathing machines, electric shocks to the heart and intensive treatments like that?
2. What are your wishes about treatment for potentially reversible conditions (such as a lung infection)?
3. What are your thoughts about being kept comfortable and letting your disease take its course naturally?

When did the discussion or discussions to decide on your DNR status occur with your health care provider?

Do you recall what triggered the initiation of the DNR discussion?

How emotionally distressing was the DNR discussion for you?

1. Not at all
2. A little
3. A moderate amount
4. A great amount

Prompt:* Was there anything specific about your discussion that was distressing?*

Talking about DNR can be very uncomfortable for patients, what can we do to make it easier?

What are your specific concerns or worries about deciding on a Do Not Resuscitate Status?

Do you believe that it is possible to change a DNR status?

Are you worried that the level of the medical care or attention you receive may be affected by deciding on a Do Not Resuscitate status?

Who have you ever discussed your resuscitation status with? 

1. Surgeon who biopsied or did surgery
2. Medical Oncologist who decided about your chemotherapy
3. Radiation Oncologist who decided about your radiation
4. Medical Resident
5. Nurse
6. Psychosocial Counselor
7. Family physician
8. Palliative Care Physician
9. Medical Student
10. Other

Prior to your first DNR discussion with a health care provider, had you already made your own decisions concerning resuscitation status?

Have you ever signed any documents specifying your DNR status? (Living will, advanced care plan, health care directive)

Who do think is the most appropriate person to discuss your DNR status with you? 

1. Surgeon who biopsied or did surgery
2. Medical Oncologist who decided about your chemotherapy
3. Radiation Oncologist who decided about your radiation
4. Medical Resident
5. Nurse
6. Psychosocial Counselor
7. Family physician
8. Palliative Care Physician
9. Medical Student
10. Other

Prompt: *I am interested in knowing why you chose this person as being most appropriate to discuss DNR with you. Can you tell me more about your choice?*

When do you feel is the most appropriate time to discuss DNR?

1. Never
2. At the time of initial diagnosis
3. When first time told of poor prognosis (small or no chance of cure)
4. On a follow-up visit after previously been told of the poor prognosis
5. When discussing referral to a palliative care program
6. When pain or other symptoms are not responding to medical management
7. When hospital/palliative care admission is required
8. Should be discussed only when death imminent
9. Other

Prompt:* I am interested in knowing why you chose this time to discuss DNR as most appropriate? *

Who should be present in the discussion of DNR? 

1. Physician
2. Single family member or support person
3. Multiple family members or support persons
4. Nurse
5. Psychosocial counselor
6. Others

Prompt: *I am interested in knowing why you chose this individual (these individuals) to be present in the discussion of DNR.*

Have you and your family members or friends talked about what your thoughts and feelings are in regards to DNR?

1. Never
2. Rarely
3. Sometimes
4. Frequently
5. Always

What is the extent to which your family members or friends assist you in coping with your cancer?

1. Never assist me
2. Rarely
3. Sometimes
4. Frequently
5. Always

What do you think is the best environment to discuss DNR? (Circle all that apply)

1. Clinic
2. Hospital
3. Home
4. Other

Prompt: *Can you tell me why this is the best environment to discuss DNR?*

What is your cultural or ethnic background? For example, what part of the world do your ancestors come from?

Prompt: *What influence does your cultural background have on your decisions and views concerning DNR?*

Prompt: *What influence do your religious beliefs have on your decisions and views concerning DNR?*

How emotionally distressing was today’s interview process and discussion for you?

1. Not at all
2. A little
3. A moderate amount
4. A great amount

Prompt: *Do you have any suggestions on how to reduce any distress associated with completing a survey such as this?*

Please provide any additional comments or suggestions you have in regards to DNR discussions that might not have been addressed by this survey.

Appendix 2: Family/Caregiver Survey                   

Prompt*: Today we’re going to be talking about medical issues such as advance care planning and resuscitation status, which can be a tiring and emotional experience for some. May I remind you that you are free to decline to answer any or all questions if you wish? Over the course of the interview, you and/or the patient may become fatigued or feel too unwell to continue the interview. If this happens, you and/or the patient can request the interviewer to stop the interview at any time. The interviewer will then invite you and/or the patient to continue the interview at another time or date that is convenient for you and/or the patient. Please ask for clarification if any questions are difficult to understand. For the purpose of this survey, such terms as “resuscitation status,” “Do Not Resuscitate or DNR order” and “Advance Care Plan or ACP level” may be used frequently and interchangeably.*

What is your understanding of the patient’s current DNR status?

How would you rate your understanding of DNR? (Circle one)

1. Lacking in some areas
2. Adequate
3. Good
4. Excellent
5. Extremely limited

How distressing do you think the DNR discussion was for the patient?

1. Not at all
2. A little
3. A moderate amount
4. A great amount

Who first brought up the issue of DNR for discussion? 

1. Medical Oncologist who decided about chemotherapy
2. Radiation Oncologist who decided about radiation
3. Nurse
4. Psychosocial Counsellor
5. Family physician
6. Medical Resident
7. Medical Student
8. Not sure
9. Surgeon who biopsied or did surgery
10. Other

Who do think is the most appropriate person to discuss DNR? 

1. Surgeon who biopsied or did surgery
2. Medical Oncologist who decided about chemotherapy
3. Radiation Oncologist who decided about radiation
4. Nurse
5. Psychosocial Counsellor
6. Family physician
7. Medical Resident
8. Medical Student
9. Not sure
10. Other

Prompt: *I am interested in knowing why you chose this person as being most appropriate to discuss DNR with. Can you tell me more about your choice?*

When do you think is the most appropriate time to discuss DNR? 

1.  At the time of initial diagnosis
2. When first time told of poor prognosis (small or no chance of cure)
3. On a follow-up visit after previously been told of the poor prognosis
4. When discussing referral to a palliative care program
5. When pain or other symptoms are not responding to medical management
6. When hospital/palliative care admission is required
7. Should be discussed only when death imminent
8. Other
9. Never

Prompt: *I am interested in knowing why you chose this time as the most appropriate for DNR discussion. *

Prompt: *I am interested in knowing why you chose these individual/these individuals to be present in the discussion of DNR.*

What would be of concern to you when/if discussing DNR?

Talking about DNR can be very uncomfortable for patient and families. What can we do to make it easier*?*

What is the best environment in which to discuss DNR? (Circle one or all that apply)

1. Clinic
2. Hospital
3. Home
4. Other

Prompt:* Can you tell me why this is the best environment in which to discuss DNR?*

Appendix 3. Healthcare Provider Survey

Are you comfortable when discussing DNR with your patients in given circumstances?

a. Yes

b. No

Prompt: *Please explain the reasons for your choice.*

Do you think we should revisit the current practice of DNR at CCMB?

a. Yes

b. No              

Prompt: *Please explain the reasons for your choice.*

Do you think your patients understand DNR when discussed? 

1. Never
2. Rarely
3. Sometimes
4. Frequently

Prompt: *Please explain how you know your patient understands or does not understand DNR when discussed.*

How often do you have a DNR discussion with a patient in your practice?

1. Once a week
2. Once every two weeks
3. Once a month
4. Other

Do you think you have sufficient time to discuss DNR with your patients in your practice environment?

1. Never
2. Rarely
3. Sometimes
4. Usually
5. Always

Who do think is the most appropriate person with whom to discuss DNR? (Circle One)

1. Surgeon
2. Medical Oncologist
3. Radiation Oncologist
4. Nurse
5. Psychosocial Counsellor
6. Family physician
7. Other

Prompt: *I am interested in knowing why you chose this person as being most appropriate with whom to discuss DNR. Can you tell me more about your choice?*

When do you think is the most appropriate time to discuss DNR? 

1. Never
2. At the time of initial diagnosis
3. When first time told of poor prognosis (small or no chance of cure)
4. On a follow-up visit after previously being told of poor prognosis
5. When discussing referral to a palliative care program
6. When pain or other symptoms are not responding to medical management
7. When hospital/palliative care admission is required
8. Should be discussed only when death imminent
9. Other

Prompt: *I am interested in knowing why you chose this time to discuss DNR? *

Do you think it is worth doing a prospective clinical trial to determine the best timing of discussing DNR?

a. Yes

b. No

Prompt: *Why or why not?*

Who should be present in the discussion of DNR? 

1. Physician
2. Single family member or support person
3. Multiple family members or support persons
4. Nurse
5. Psychosocial counsellor
6. Others

Prompt: *I am interested in knowing why you chose these individual/these individuals to be present in the discussion of DNR.*

For you, what patient factors add complexity to DNR discussions? (Age, religion, language, ethnicity, family, socioeconomic status, timing of diagnosis, sudden progression of disease, sudden worsening of symptoms, etc.)

How often do you think patients find DNR discussions distressing

1. Never
2. Rarely
3. Sometimes
4. Frequently
5. Always

Prompt: *Talking about DNR can be very uncomfortable for patients; do you have any suggestions on how to make it easier for them?*

What is the best environment to discuss DNR? 

1. Clinic
2. Hospital
3. Patients home

Prompt: *Can you tell me why this is the best environment to discuss DNR?*

How long does the discussion of DNR usually take in your practice?

Do you think we need a structured information sheet and script to discuss DNR

a. Yes

b. No

Prompt: *Please explain reasons for your choice.*

Would you like additional resources or training for DNR discussions?

a. Yes

b. No

Prompt: *Please explain reasons for your choice.*

What suggestions/ideas do you have to improve DNR discussions at CCMB?
